# P-833. The trends of hospital-onset bacteremia at a tertiary care center and the impact of the establishment of infectious diseases department on its incidence: a 10-year longitudinal study

**DOI:** 10.1093/ofid/ofae631.1025

**Published:** 2025-01-29

**Authors:** Yuya Kawamoto, Yohei Doi, Hitoshi Honda

**Affiliations:** Fujita Health University School of Medicine, Toyoake, Aichi, Japan; University of Pittsburgh, Toyoake, Aichi, Japan; Fujita Health University School of Medicine, Toyoake, Aichi, Japan

## Abstract

**Background:**

Hospital-onset bacteremia (HOB) is defined as the onset of bacteremia occurring three or more days after hospitalization. The incidence density of HOB has recently been used to assess the quality of overall hospital infection control practice in lieu of the central line-associated bloodstream infection incidence rate since the HOB incidence might better predict the length of stay, hospital costs, and mortality rates. At the study institution, Infectious Diseases (ID) department was established in April 2018. The present study aims to investigate the incidence density of HOB prior to and after the establishment of ID department.

Change and trend of the incidence density of HOB before and after the establishment of ID department as determined by ITSA.

**Figure:**
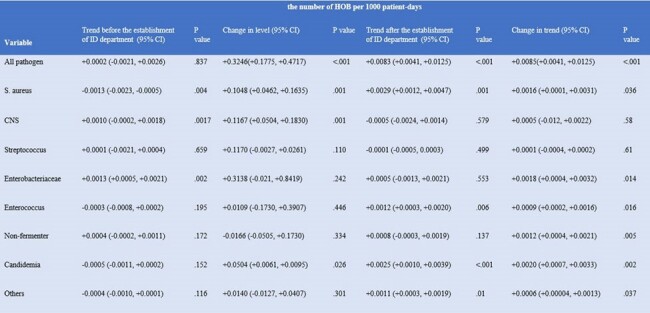

**Methods:**

We investigated the monthly incidence density of HOB per 1,000 patient-days from 2013 through 2023 at Fujita Health University Hospital. The trends of overall incidence of HOB and HOB by causative pathogens group, including *Streptococcus* species (spp), *Staphylococcus aureus*, Coagulase-negative staphylococci (CNS), *Enterococcus* spp., Enterobacterales, and *Candida* spp. were tracked. Changes in the trend of HOB before (from July 2013 through March 2018) and after (from April 2018 through June 2023) the establishment of ID department were evaluated. Interrupted time series analysis was used for statistical analyses.

Change and trend of the incidence density of HOB before and after the establishment of ID department as determined by ITSA.

**Figure:**
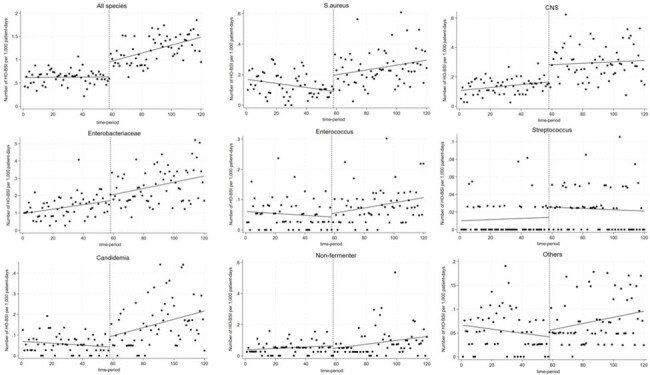

**Results:**

In total, 4,422 HOB events were identified during the study period. The overall incidence density of HOB was 0.94 per 1,000 patient-days. Comparison of the incidence of HOB between the period before and after the establishment of ID department revealed an immediate increase in the incidence of overall HOB (+0.3246; P< .001 for change in level) and HOB caused by *S. aureus* (+0.10; P< 0.001 for change in level), CNS (+0.12; P< 0.001 for change in level), and *Candida* spp. (+0.05; P=0.03 for change in level). A significant trend increase in the incidence of HOB was also observed in HOB due to *S. aureus* (+0.002; P< .001 for change in trend) *Candida* spp. (+0.002; P=0.002 for change in trend), and Enterobacterales (+0.002; P=0.002 for change in trend).

**Conclusion:**

The overall incidence density of HOB has increased significantly in the last decade, and the trend was steeper after the establishment of ID department. Improvement in ID clinical practice may contribute to increasing the detection of HOB.

**Disclosures:**

**Yohei Doi, MD, PHD**, AbbVie: Honoraria|Entasis: Grant/Research Support|Gilead: Advisor/Consultant|GSK: Advisor/Consultant|Meiji Seika: Advisor/Consultant|Moderna: Advisor/Consultant|Pfizer: Advisor/Consultant|Shionogi: Advisor/Consultant|Shionogi: Honoraria **Hitoshi Honda, MD**, Moderna Inc: Honoraria|Pfizer: Honoraria|Shionogi: Honoraria

